# Equine placental extract supplement as a night barking remedy in dogs with cognitive dysfunction syndrome

**DOI:** 10.1002/vms3.893

**Published:** 2022-08-03

**Authors:** Tatsuya Amano, Takashi Ikeda, Makiko Yamaguchi, Nobuhisa Kakehi, Keizo Hanada, Tsuyuko Watanabe, Kentarou Tahara, Eiichi Hirano

**Affiliations:** ^1^ Amano Animal Hospital Okayama Japan; ^2^ Momo Animal Hospital Kobe Japan; ^3^ Rui Animal Hospital Tokoname Japan; ^4^ Domestic Sales Department Japan Bio Products Co., Ltd. Shibuya Japan; ^5^ Medical Affairs Department Japan Bio Products Co., Ltd. Shibuya Japan; ^6^ Research Institute Japan Bio Products Co., Ltd. Kurume Japan

**Keywords:** barking at night, canine cognitive dysfunction syndrome, placental extract, senior pet care, supplement

## Abstract

With the aging of pet dogs, there has been an increasing trend in senility‐related diseases; additionally, cognitive disorders accompanied by abnormal behaviours are a major burden for owners. Recently, there have been a series of consultations regarding the fact that night barking, which is an abnormal behaviour, remarkably interferes with the owner's sleep and adversely affects the owner's quality of life. However, there has been no effective solution to this problem. In this study, three aged pet dogs diagnosed with dementia were administered an equine placental extract (eqPE) as pet supplement, which has been shown in laboratory models to improve cognitive function. Consequently, night barking ceased 1 week after the administration of eqPE in case 2 and it was observed to decrease in the other two dogs. Furthermore, night barking disappeared 2 and 3 weeks after the administration of eqPE in cases 1 and 3, respectively. No recurrence or exacerbation of night barking was observed in the three cases treated with the eqPE, and no adverse events were observed. These results suggest that eqPE may be useful for improving night barking in pet dogs with dementia, and it is expected to be a new treatment method.

## Graphical abstract

An equine placenta extract (eqPE) as pet supplement could ameliorate CDS‐associated night barking in three aged pet dogs diagnosed with dementia.

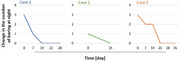

## 1 INTRODUCTION

Canine cognitive dysfunction syndrome (CDS) is a major neurobehavioural syndrome in senior dogs (Neilson et al., 2001), and is estimated to affect approximately 15 million and >30 million dogs in the United States and Europe, respectively (Bosch et al., 2012). The prevalence of CDS can be observed in up to 60% of dogs older than 11 years of age (Fast et al., 2013). The signs of behavioural dysfunction correlate with neuropathological findings, such as cortical atrophy (Borras et al., 1999), cerebral amyloid angiopathy (Su et al., 2005), and ventricular enlargement (Nakayama et al., 1991) in dogs. Besides, it causes behavioural deficits in spatial awareness, social interaction, sleeping patterns, house training, memory, and learning (Landsberg, 2005).

There has been a mutually beneficial partnership between humans and dogs for more than 100,000 years (Vila et al., 1997). Health disorders in dogs have been reported to have welfare implications for pet dogs and financial and emotional implications for owners (Nakano et al., 2019). Dog barking is a problem in several cities worldwide. In particular, several individuals find it disturbing and experience psychological distress when dogs bark at night. The results of a questionnaire survey denoted that 545 (75%) of 727 valid responses indicated that the responders experienced night barking of dogs, which was indicated to be more unpleasant than all other noises such as sounds of a lawn mower, children shouting, baby crying, motorbike revving, and skill saw (Flint et al., 2014). There has been an increase in consultations from owners whose dogs have CDS due to ageing and the associated implication of night barking. Their main concern was that the owner and his family, who live together, were intermittently disturbed by the night barking and experienced sleep deprivation.

Recent studies and clinical trials have investigated a beneficial therapy using nutritional supplements, such as antioxidants, amino acids, and vitamins, for improving the progression of cognitive dysfunction in dogs (Araujo et al., 2005, 2012; Katina et al., 2016; Milgram et al., 2004; Pan et al., 2018). For example, dogs fed a high‐quality diet designed for their age and size were approximately three times less likely to develop cognitive dysfunction than dogs fed a diet of low‐quality commercial foods or table scraps (Katina et al., 2016). Moreover, multiple active agents have been demonstrated to be more effective than individual agents (Araujo et al., 2005; Milgram et al., 2004; Pan et al., 2018).

Placental extracts derived from domestic animals, such as pigs, are obtained by enzymatically degrading the placenta. The extracts have multiple functions, including antioxidative activity, and contain multiple antioxidants, including amino acids (tryptophan, phenylalanine, and tyrosine) and uracil (Togashi et al., 2000; Watanabe et al., 2002). However, there are few case reports and studies on night barking in senior dogs with dementia, and new treatment methods need to be established. Considering the characteristics of placental extract, it is a perfect supplement and has potential as a new treatment for night barking in senior dogs with dementia.

Recently, we received consultations for three cases in which the owners were severely sleep‐deprived and their quality of life was worsened by their senior dogs with CDS frequently barking at night. Placental extracts have been reported to improve cognitive function in an Alzheimer's disease mouse model (de Toledo, et al., 2021; Kogure & Tohda, 2017) and aged mice (Yamauchi et al., 2019). Herein, we describe the administration of equine placental extract (eqPE) as a pet supplement to improve the cognitive function of these three dogs and the corresponding changes in their night barking.

## 2 CASE DESCRIPTION

### 2.1 Case 1

**FIGURE 1:**
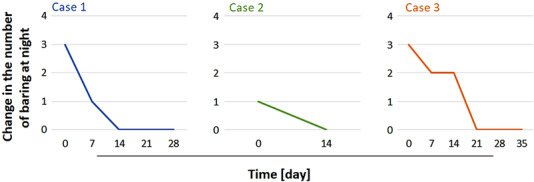
Changes in the number of barking at night of aged dogs with dementia treated with eqPE. The eqPE was administered for 21, 14, and 35 days and cases 1, 2, and 3, respectively. During each period, the number of barks at night was not observed in all cases.

A 14‐year‐old, 9.1‐kg, non‐castrated male Shiba dog was diagnosed with dementia. Thyroid hormone (Levotiron, 0.36 mg, orally, once daily, Abdi Ibrahim), donepezil hydrochloride (Aricept, 1.27 mg, orally, once daily, NIPRO), and diazepam (Cercine, 10 mg, orally, as needed, Takeda Pharmaceutical Co., Ltd.) were administered. To evaluate the number of night barks in this senior dog, owners recorded it at home. Assessment of the duration of each night's barking was abandoned owing to the inability to accurately measure the time per night barking, as well as concerns of disrupting the owner's sleep. Data were collected and tabulated regularly by veterinarians whenever the owners visited veterinary clinics. The dog was observed to squeal every few hours, not only at night but also during the day, and the demand barking was high. Other observations were that the dog heavily breathed and barked loudly when required. The dog was also a nuisance to its neighbours due to its constant barking; consequently, the dog's residence was shifted from outside to inside the house. Moreover, the dog did not respond to humans or other animals. It was assumed that the severe whimpering across the day and night was due to severe cognitive decline, and donepezil hydrochloride alone was insufficient to improve the dog's symptoms. Given that the dog had already received thyroid hormone, diazepam, and other medications, besides donepezil hydrochloride, administering further medications was halted to reduce the burden. Furthermore, since the owner preferred the use of supplements over pharmaceuticals, we decided to use some supplements. Subsequently, eqPE was selected because some owners reported improved cognitive function in their senior dogs. One week after eqPE (authors affiliation) was administered (2 ml/dog, orally, once daily), the daytime squeal disappeared. In the evening, no squeal was observed after supper up to approximately 3:00 AM. From approximately 3 AM to 6 AM, it was confirmed that excretion and urination caused little noise by the dog every 30 or 60 min. Other findings included a decrease in laboured breathing beginning 4 days after eqPE administration. No change in reaction to humans or other animals was observed compared to that before eqPE administration. From the next week, the dose of eqPE was reduced to once every 2 days, and the dog was observed for 1 week. Subsequently, no squealing was observed at night or during the day. Furthermore, the dog's laboured breathing had almost disappeared, and when breathing became laboured, drinking water improved breathing. The dog also slept for most of the day and night and was quieter than during the first week after eqPE administration (Figure 1). The reaction to humans and other animals remained unchanged. After 3 weeks of eqPE administration under the same conditions, no squeal was observed during the day and night (Figure 1). Four weeks after administration of eqPE, the dog was observed to sleep for most of the day and night, not whimper at night, and remain quiet even when not sleeping. These parameters were found to be the same as those in the third week after eqPE administration (Figure 1). However, there was no change in the dog's reaction to humans and other animals even after 3 weeks of eqPE administration. No adverse events were observed during the administration of eqPE.

### 2.2 Case 2

A 17‐year‐old, 3.0‐kg, castrated male Yorkshire terrier dog was diagnosed with dementia. The same method as that in Case 1 was used to evaluate the number of night barks in this dog. This dog was confirmed to squeal once every night for approximately 1 h. Although night barking in this dog was infrequent, it continued for approximately 1 h. Other findings in this dog were that its reaction to its surroundings was sluggish, and it was only responsive to relatively strong stimuli, such as loud noise. Furthermore, the dog wandered and circled, and when wandering in the middle of the night, the dog would lose its direction. Moreover, this dog did not want to walk. One week after eqPE administration (2 ml/dog, orally, once daily), night barking disappeared (Figure 1). Night barking was not observed for another week after eqPE administration under the same conditions (Figure 1). Other results showed that this dog's reaction to its surroundings improved to a normal level compared to that before eqPE administration. Moreover, the dog slept soundly at night and for most of the day. The dog also began walking and no adverse events due to administration were observed.

### 2.3 Case 3

A 14‐year‐old, 10.9‐kg, spayed female Shiba dog had been diagnosed with dementia. Ursodeoxycholic acid (ursodeoxycholic acid, 100 mg, orally, twice daily; NIHON GENERIC Co., Ltd.) was administered. To evaluate the number of night barks, the same method as that in Case 1 was used. This dog was confirmed to have night barking approximately three times at night (Figure 1). Other findings in this dog were normal reaction to its surroundings and no wandering or loss of orientation. One week after eqPE administration (2 ml/dog, orally, once every 3 days), night barking reduced (Figure 1). No other findings were observed in the first week after eqPE administration. Subsequently, 2 weeks after eqPE administration under the same conditions, night barking reduced (Figure 1). No other specific findings were observed. Subsequent eqPE administration for 4 weeks under the same conditions resulted in the cessation of night barking (Figure 1). No other findings were observed. Furthermore, no adverse events caused by eqPE administration were observed.

## 3 DISCUSSION

In this case report, we observed that eqPE administration improved night barking in three senior dogs with CDS. These results support the use of eqPE for improving cognitive health in senior dogs, especially those that bark at night due to CDS. Moreover, this is the first published case report to demonstrate the cognitive benefits of a therapeutic drug for night barking in senior dogs with CDS. Besides, no adverse effects due to the eqPE administration were observed in any of the three dogs. The eqPE is extracted from the placentas of horses through an enzymatic digestion process derived from livestock, and its components are mostly amino acids and small peptides (Han et al., 2018; Togashi et al., 2000; Watanabe et al., 2002). Therefore, they are unlikely to contain substances that may be antigenic or cause other problems. Furthermore, the raw material, equine placenta, is obtained from horses under strict breeding management to prevent contamination with hormones, tranquillisers, and doping agents. Therefore, the possibility of side effects from eqPE administration is presumed to be very low.

As mentioned above, although it can be logically inferred that the main components of eqPE are amino acids and low‐molecular‐weight peptides, it is difficult to identify all of them. None of the active components in eqPE have yet been identified. However, at the basic research level, the active components of placental extract have been identified as heptapeptides, which have been shown to promote dendrite elongation and improve cognitive dysfunction in a mouse model of Alzheimer's disease (Tohda et al., 2021). Other studies have shown that hydroxyproline‐containing dipeptides identified from placental extracts efficiently promote mucin and tear fluid secretion in the rabbit ocular surface epithelium (Nakamura et al., 2015) and promote corneal epithelial cell growth, cell migration, and wound healing in the corneal epithelium (Nagata et al., 2015). Moreover, glycine and leucine dipeptides have been reported to improve skin moisturisation (Park et al., 2017) and elasticity in hairless mice. Leucine and glycine dipeptides reduce physical fatigue in mice by enhancing the dopaminergic system (Han et al., 2018). Therefore, based on the above reports, the active ingredient of eqPE may be a peptide. Contrastingly, some studies have reported that placental extract was effective at doses higher than 1000 mg/kg (Kogure & Tohda, 2017; Takuma et al., 2012; Yamauchi et al., 2019). Branched‐chain amino acid an amino acid preparation, has been reported to be effective at doses as high as 1000 mg/kg or more in patients with liver diseases (Marchesini et al., 2005). From these findings, it cannot be excluded that the active component of eqPE may be amino acids.

Studies on supplements to improve cognitive function in older dogs have been conducted (Araujo et al., 2005, 2012; Milgram et al., 2004; Pan et al., 2018). These studies typically evaluated the effects of supplements on discriminative learning ability and did not evaluate night barking in dogs. It is likely that the number of cases was too small or no cases used night barking as an outcome. To our knowledge, there are no studies evaluating night barking as an outcome of CDS in older dogs. Therefore, although it is not possible to compare the potency of eqPE and supplement prior studies at this time, it may be possible to speculate on its utility. The supplements used in previous studies were mixtures of specific useful ingredients or nutrients, such as vitamins and antioxidants. In contrast, eqPE is a multifunctional supplement with antioxidant properties rich in amino acids. The eqPE with its multifunctional potential properties may be more useful as a multifaceted response supplement for older dogs with CDS with a variety of symptoms. The three cases in this study had severe night barking, which negatively affected the owners’ quality of life and may have been relatively rare. However, the ongoing increase in the number of CDS consultations regarding the correction of night barking in older dogs suggests that it may previously have been an overlooked symptom. Therefore, further epidemiological studies on the onset of night barking and CDS are warranted.

In the present study, night barking in Case 1 was caused not only by CDS, but also by anxiety and demand for something specific. The anxiety‐induced night barking did not improve after changing the dog from an outdoor to an indoor pet. Although the improvement of the rearing environment did not improve night barking in both the day and night time anxiety‐induced barking, it disappeared after eqPE administration. This suggests that eqPE administration may be effective for anxiety‐induced night barking and its influence may be mediated through the central nervous system. This finding is consistent with the hypothesis that placental extract prevents hippocampal atrophy in menopausal model mice and ameliorates cognitive function in chronic restraint stress in ovariectomised mice model with Alzheimer's disease, and older mice (de Toledo, et al., 2021; Kogure & Tohda, 2017; Takuma et al., 2012; Yamauchi et al., 2019). Moreover, the influence was observed within a relatively short period of 1 week after the eqPE administration indicating that it has a fast onset of effect. To the best of our knowledge, this is an unparalleled study. Demand‐induced night barking was remarkably reduced after 1 week of eqPE administration and disappeared after 2 weeks of administration. These results suggest that eqPE administration is effective against night barking caused by multiple factors, and it is inferred that it has anxiolytic effects.

A decrease in response to the surroundings was observed in Case 2, and it was presumed that this included night barking caused by anxiety and CDS. Although the initial level of night barking was one rank lower than in the other two cases, improvement in night barking was observed within 1 week after eqPE administration, as in the other two cases. Therefore, it was concluded that eqPE was effective in improving night barking for multiple reasons. Furthermore, after eqPE administration, the dog in Case 2 was observed to sleep better at night. These results suggest that eqPE has an effect on dog sleep. However, it is unclear whether improved sleep quality was an effect of eqPE in this case. In human studies, it has been reported that porcine placental extract does not affect the duration of sleep, but it improves the quality of sleep (Nagase et al., 2020); therefore, it is assumed that eqPE had a similar effect in dogs. Currently, we are conducting research using mouse models with sleep disorders to elucidate the effects and mechanism of eqPE.

In Case 3, it took longer to improve night barking by eqPE administration than in the other two cases. The time lag in the improvement of night barking by eqPE administration was 1 week and two times longer than that in Case 1 and 2 weeks longer than that in Case 2. This time lag may be explained by the differences in the frequency of eqPE administration in each case. In cases 1 and 2, eqPE was initially administered daily, whereas in Case 3, it was administered once every 3 days. This suggests that the time lag in the improvement of night barking was due to the lower total dosage of the supplement. Previous studies have shown that placental extracts have concentration‐dependent pharmacological effects (Kogure & Tohda, 2017; Yamauchi et al., 2019).

In Case 1, donepezil hydrochloride had already been administered as a dementia drug, but in the other cases, no anti‐dementia drug was administered. The administration of eqPE resulted in the disappearance of all cases of dementia‐induced night barking. From these results, it is presumed that eqPE administration may contribute to the decline in night barking in dogs with CDS. Several studies have indicated that placental extracts contribute to improved cognitive function. Placental extracts have been shown to suppress hippocampal atrophy in menopausal mice model and improve cognitive function in model mice with Alzheimer's disease (de Toledo, et al., 2021; Kogure & Tohda, 2017; Takuma et al., 2012). Porcine placental extract ameliorated the age‐associated decrease in memory function in mice. Moreover, its effect on the hippocampus of aged mice may be mediated via the upregulation of *Early growth response protein 1* (*Egr1*), *Growth Arrest and DNA‐Damage‐Inducible, Beta* (*GADD45b*), *NGFI‐A Binding Protein 2* (*NAB2*), and *Vascular Endothelial Growth Factor A* (*VEGF‐A*) genes (Yamauchi et al., 2019). Moreover, we found that eqPE prevented the onset of memory dysfunction in 5XFAD mice, a model of Alzheimer's disease, and elevated neurogenesis and dendrite density after Aβ treatment (de Toledo, et al., 2021). Thus, our speculation is supported by the aforementioned reports. Furthermore, we speculated that the multifunctional actions of eqPE such as protecting brain tissue and upregulation of genes related to cognitive function and enhancing neurogenesis similar to the aforementioned research results may have contributed to the improvement of night barking due to dementia in all the three dogs in this study.

In conclusion, this case report demonstrates that eqPE could ameliorate CDS‐associated night barking in dogs. Future long‐term follow‐up is recommended in these dogs to observe symptoms and to continue long‐term administration of supplements. Moreover, systematic clinical trials on the efficacy, safety, and mode of action of eqPE in dogs with CDS are recommended.

## CONFLICT OF INTERESTS

Tatsuya Amano, Takashi Ikeda, and Yamaguchi Makiko declare no conflict of interest. Nobuhisa Kakehi, Keizo Hanada, Tsuyuko Watanabe, Kentaro Tahara, and Eiichi Hirano are employees of the Japan Bio Products Co., Ltd.

## ETHICAL STATEMENT

The authors confirm that the ethical policies of the journal, as noted on the journal's author guidelines page, have been adhered to and that no ethical approval was required for this case report.

## AUTHOR CONTRIBUTIONS

Tatsuya Amano, Takashi Ikeda, and Makiko Yamaguchi conceived and designed the clinical study and analysed and interpreted the data. Nobuhisa Kakehi, Keizo Hanada, Tsuyuko Watanabe, and Kentarou Tahara analysed and interpreted the data.

Eiichi Hirano analysed and interpreted the data and wrote the manuscript.

### PEER REVIEW

The peer review history for this article is available at https://publons.com/publon/10.1002/vms3.893

## Data Availability

The data that support the findings of this study are available from the corresponding author upon reasonable request.
